# Investigating the relationship between corruption and health system outcomes in Central and Eastern Europe

**DOI:** 10.1093/eurpub/ckae218

**Published:** 2025-10-22

**Authors:** Petra Varkonyi, Martin McKee, Darius Erlangga, Dina Balabanova

**Affiliations:** London School of Hygiene & Tropical Medicine, London, United Kingdom; Department of Global Health and Development; London School of Hygiene & Tropical Medicine, London, United Kingdom; Department of Health Services Research and Policy; London School of Hygiene & Tropical Medicine, London, United Kingdom; London School of Hygiene & Tropical Medicine, London, United Kingdom

## Abstract

Corruption in the health sector wastes precious resources and threatens progress to universal health coverage (UHC). Countries of Central and Eastern Europe (CEE) have undertaken extensive health system reforms, many including measures to tackle informal payments and other forms of corruption. We asked whether there is evidence linking perceived corruption in twelve CEE countries between 2012 and 2020 with two health system-related outcomes, out-of-pocket payments and avoidable mortality. We analyse panel data and adjust for national characteristics in the political, economic, and social domains. We find a significant association between perceived corruption and health system outcomes after accounting for time-variant and in-variant factors using fixed-effects analysis. The political, economic, and social determinants of health, including quality of government, public health expenditure, national income, inequality, and urbanisation have stronger associations with each health system outcome. The results suggest that while corruption is detrimental to population health, it is one of a number of political and economic factors that must be addressed to accelerate progress towards equitable and universal health coverage. The direct and indirect effects of corruption on factors outside the health system that impact health outcomes cannot be ignored. Intersectional and global anti-corruption, accountability, and transparency initiatives remain a critical priority for achieving UHC in CEE and ensuring health systems keep pace with economic growth and population expectations.

## Introduction

Governments have committed to achieving universal health coverage (UHC), as set out in Sustainable Development Goal 3 [1]. Progress is, however, threatened by widespread corruption in many health systems, especially where governance is weak [2]. This threat was explicitly recognised in the political declarations following the UN Assembly High-level Meetings on UHC in 2019 and in 2023 [3]. The Global Network on Anti-Corruption, Transparency, and Accountability (GNACTA) seeks to increase understanding of how health sector corruption impacts health, and how to combat it by promoting good governance and health system strengthening [4]. 

Hutchinson *et al.* define corruption as “the abuse or complicity in abuse, of public or private position, power or authority to benefit oneself, a group, an organisation or others close to oneself in a way which diverts institutions from their core aims; where the benefits may be financial, material or non-material” [5]. This abuse damages trust, hinders economic progress, undermines democratic values, and exacerbates societal disparities.

Annual financial losses to public health budgets attributable to corruption in Europe were estimated at €56 billion in 2018 [6] and there is growing research linking corruption to worse health—including lower life expectancy, higher mortality, reduced immunisation rates, higher chronic disease burden, and poor mental health [7, 8]. The effects of corruption manifest through various mechanisms. First, where resources are already scarce, corruption hampers economic growth, reducing household income and associated health determinants [9]. Second, funds lost to corruption cannot be spent on improving the wider determinants of health. This affects countries at all levels of development, such as the procurement scandals during the pandemic in some high-income countries [10]. Finally, corruption reduces trust in health systems [11].

The health sector is particularly susceptible to corruption as interactions often take place in private and are characterized by information asymmetries [12]. Corruption can take many forms, including bribery, ghost workers, and procurement irregularities, but one form, informal payment to health workers, is prevalent in Central and Eastern Europe (CEE). Strategies emphasising transparency and accountability, community monitoring, and controls on insurance fraud have shown promise in diminishing corruption [2].

Measures of corruption can be captured in several ways, including surveys of perceptions and household and public expenditure, and reviews of control systems [2]. By virtue of its illicit nature, those engaged in corrupt activities have powerful incentives to conceal it [13]. Transparency International publishes annual measures of their Corruption Perception Index (CPI), a widely used global corruption measure ranking 180 countries, based on expert and business leader surveys [14]. The Special Eurobarometer 397 report found that healthcare corruption correlates with broader public sector corruption perceptions [15]. A meta-analysis including 42 studies of corruption and health found that most used the CPI [16], shown to correlate well with other indicators [17].

This paper arose following news from Transparency International that its CPI had either failed to improve or worsened in these countries over the past decade [14]. Their index is derived from expert perceptions of corruption levels but it is consistent with polling undertaken for the 2022 Special Eurobarometer, which reported that 68% of EU citizens perceive corruption as widespread in their country [18], with Kartal drawing particular attention to the problem in CEE [19]. This situation co-exists with a common perception that countries in this region have achieved, or are close to achieving, universal coverage, with health sector corruption much less of a problem than in the 1990s. However, to our knowledge, no studies have examined the association between corruption and health system outcomes across time in CEE.

This paper seeks to understand the complex relationship between corruption and health system outcomes in CEE. Despite the fact that relevant data are available, few studies have examined this question in Europe [8], and what exists is often small-scale studies in single countries [20]. To address this gap, we test the hypothesis that perceived corruption in CEE will be associated with greater mortality from conditions amenable to healthcare and reduced financial protection as captured by increased out-of-pocket (OOP) payments. This hypothesis is compatible with analyses linking corruption with mortality [21].

We aim to investigate the association between corruption and two health system outcomes in 12 CEE countries—Bulgaria, Croatia, Czech Republic, Estonia, Hungary, Latvia, Lithuania, Poland, Romania, Serbia, Slovak Republic, and Slovenia—all currently European Union members or candidate countries. Our comparative analysis seeks to control for country-specific political, economic, and social factors between 2012 and 2020.

## Methods

Our conceptual model seeks to ascertain any association between corruption and two core health system functions, improving health (incorporating access and quality) [22] and fair financing [23] (Supplementary Appendix Table S1). We used the CPI as the independent variable to approximate corruption levels. The CPI ranks countries and territories globally by their perceived levels of public sector corruption on a scale of 0–100, where 0 indicates a highly corrupt public sector and 100 an immaculate one [14]. For the dependent variables, we capture access and quality using Eurostat data on avoidable mortality [24]. Avoidable mortality is a measure of deaths that should not occur in the presence of timely and effective prevention and care [25]. It includes preventable and treatable causes of mortality, the former defined as mortality avoidable through effective public health and primary prevention interventions, and the latter as mortality, which can largely be avoided with health care [24]. We capture fair financing using data on the share of OOP expenses as a percentage of total health expenditure, sourced from the World Bank’s World Development Indicators [26]. OOP payments are defined as any spending incurred by a household when any member uses a health good or service to receive any type of care (preventive, curative, rehabilitative, long-term or palliative care); provided by any type of provider; for any type of disease, illness, or health condition; in any type of setting (outpatient, inpatient, at home) [26]. Given that there is no single pathway between corruption and health outcomes, we chose to explore two indicators that are reflective of a well-functioning and equitable healthcare system. OOP payments, which include informal payments, are indicative of catastrophic spending and, therefore, may be on the pathway as such payments may limit access to essential and timely health services needed to retain good health [27]. Similarly, avoidable mortality indicates a responsive and efficient healthcare system and may indicate the scope for health improvement if corrupt practices were curbed.

We sought to identify putative covariates, drawing in particular on a meta-analysis by Judge *et al.* [16] and on other research on corruption and health outcomes (Supplementary Appendix Table S2). We grouped them into three domains.

### Political domain

Encompasses political openness, structure, and government effectiveness. Judge *et al.* define political openness as the presence of liberal democracy, electoral rules, voice of citizens, political freedoms and rights, and freedom of the press, while government effectiveness captures the overall perceived quality of government actors and agencies [16]. Numerous studies have connected better governance scores with improved health outcomes [28]. We employed three indicators of the quality of governance from the V-Dem dataset, including the liberal democracy, accountability, and rule of law indices [29]. These indicators range from 0 to 1, with a higher value indicating better governance.

### Economic domain

Factors such as economic wealth, growth, openness, equity, and efficiency influence corruption and its health effects. Government health expenditure (GHE) per capita, gross domestic product (GDP) per capita, and unemployment were captured within this domain [16], for which we sourced data from the WHO’s Global Health Expenditure Database and the World Bank’s World Development Indicators [26, 30]. GDP and GHE distributions, which are skewed, were log-transformed to normalize them.

### Social domain

This involves social diversity, education level, inequality degree, social values, and religious affiliation extent [16]. We used the Gini Index—where 0 represents perfect equality, while an index of 100 implies perfect inequality—and urban population as a percentage of total population from the World Development Indicators database [26].

Our analysis proceeded as follows. First, the main variables were examined visually to identify any obvious patterns and relationships and explored further with a matrix of bivariate ordinary least squares (OLS) regressions (Supplementary Appendix Table S4). In the second stage, a panel data analysis was conducted (Table 1) to study time-related effects, accounting for autocorrelation and potential endogeneity.

**Table 1.** Fixed-effects panel data analysis of each health system outcome

	Avoidable mortality			Out-of-pocket payments		
	Effect size	95% confidence interval	*P*-value	Effect size	95% confidence interval	*P*-value
Corruption Perception Index	−2.64	[−4.68 to −0.60]	0.012	0.15	[0.00 to 0.30]	0.045
Accountability Index	−185.71	[−311.85 to −59.57]	0.004	0.65	[−8.37 to 9.66]	0.887
Rule of law	−165.71	[−321.62 to −9.79]	0.038	−2.37	[−13.61 to 8.88]	0.677
Liberal Democracy Index	476.75	[170.47 to 783.03]	0.003	3.55	[−18.43 to 25.54]	0.749
GHE per capita (current intl $)	122.79	[45.15 to 200.44]	0.002	−14.43	[−20.40 to −8.46]	0.000
GDP per capita (current intl $)	−294.04	[−418.93 to −169.14]	0.000	12.37	[2.06 to 22.69]	0.019
Gini Index	−1.50	[−5.35 to 2.34]	0.439	−0.12	[−0.39 to 0.16]	0.404
Unemployment				0.07	[−0.16 to 0.29]	0.564
% Urban population	18.16	[4.00 to 32.33]	0.013	0.33	[0.70 to 1.36]	0.531
Constant	1657.61	[888.43 to 2426.79]	0.000	−24.11	[−98.67 to 50.46]	0.522
Number of observations	106			106		

*R* ^2^	Within = 0.4635			Within = 0.4014		
	Between = 0.0509			Between = 0.2827		
	Overall = 0.0554			Overall = 0.2836		

To account for heterogeneity, both random- and fixed-effects models were estimated for each outcome using OLS. The Hausman specification test determined that fixed-effects models were more suitable for both outcomes. A fixed-effect model also addresses the risk from time-invariant or unobserved covariates such as health system features or cultural factors.

All statistical computations utilized Stata SE version 17, while plots were produced with Microsoft Excel.

## Results

### Descriptive findings

#### Avoidable mortality

Avoidable mortality rates in the CEE nations ranged from 297 to 591 per 100 000 population in 2012 and from 245 to 504 by 2019 (Supplementary Appendix Fig. S1). Although 2020 witnessed a surge in average mortality rates, likely due to the COVID-19 pandemic, the previous trend had been steadily downward. Lithuania, Latvia, Romania, and Hungary reported some of the highest rates of avoidable mortality. Conversely, Slovenia and the Czech Republic had the lowest rates within the group.

#### OOP payments

OOP payment average across the CEE nations was 26.1% in 2012, which dipped to 22.7% in 2020, albeit with substantial fluctuations across countries (Supplementary Appendix Fig. S2 and Table S3). Bulgaria, Latvia, Serbia, and Lithuania exhibited the highest OOP payment rates, with numbers surpassing the 30% mark, except Lithuania, which saw a minor decline below this percentage in 2020. Bulgaria experienced a sharp decrease in its OOP payments, plummeting from 47.8% in 2012–36.6% in 2020. The Czech Republic, Slovenia, and Croatia were the only nations falling below the 15% mark, which is recognized as the threshold below which catastrophic financial hardships caused by OOP payments become negligible [27].

### Fixed-effects panel data regression analysis

#### Avoidable mortality

A one-unit improvement in the CPI is associated with reduced avoidable mortality, with a central estimate of 2.64 per 100 000 persons. To put this in perspective, taking 2019 as the last year before the pandemic, if Serbia, the country with the lowest CPI score among our countries (36) was to achieve the same score as Denmark, the highest in the world (87) with a difference of 51 points, its avoidable mortality, all else being equal, would fall by 135 per 100 000 [95% confidence interval (CI) −238.7 to −30.6], with the central estimate taking it from almost 400–265 per 100 000, a 34% reduction.

The other coefficients in the analysis point to further potentially substantial gains. For example, a one-unit improvement in the perception of government accountability reduces the avoidable mortality rate by almost 186 deaths per 100 000 persons (95% CI −311.85 to −59.57). As expected, GDP growth is associated with lower avoidable mortality, with a 1 percentage point increase in GDP associated with a reduction in avoidable mortality by 294 deaths per 100 000 persons (95% CI −418.93 to −169.14). However, caution is needed as there are some counterintuitive results, such as the positive association between avoidable mortality and GHE and the Liberal Democracy Index.

Given the large increase in avoidable mortality in 2020, coinciding with the onset of the Covid-19 pandemic, we conducted a sensitivity analysis after excluding that year (Supplementary Appendix Table S5). Although some of the governance indicators (accountability and liberal democracy) and GHE were no longer significant, the CPI was, with a similar magnitude and in the same direction [−1.72 (95% CI −3.05 to −0.40)].

#### OOP payments

A one-unit increase in CPI is associated with a 0.15% increase in OOP payments, with the rest of the governance factors showing no significant association. GHE appears to have the largest and most highly significant effect on OOP payments; a one percentage point increase in GHE decreases OOP payments by 14.4% (95% CI −20.40 to −8.46). On the other hand, a unit increase in GDP correlates with a 12.4% increase in OOP payments, but it is important to note that these data do not distinguish formal and informal payments. There are no significant associations with inequality, unemployment, and urbanization.

The within *R*^2^ value surpasses the total *R*^2^ value in every model for each outcome. This suggests that when it comes to explaining variations in the outcome variables, fluctuations within each country (over time) play a more prominent role than the combined effect of all independent variables (both those that change over time and those that remain constant). The results emphasize the weight of individual country differences (or heterogeneity) in predicting the outcomes. This finding highlights the significant role of factors that change over time. Time-invariant factors seem to be less influential in explaining variations in the outcome compared to factors that do change over time.

## Discussion

This paper sought to examine the influence of corruption on health in 12 CEE countries between 2012 and 2020. The region has been marked by robust economic growth coupled with challenges such as pronounced inequalities, notable corruption, and governance issues [19]. All countries have achieved formally universal coverage using different combinations of social insurance and tax-based funding. However, a significant concern remains the dependence on OOP health payments, especially impacting the disadvantaged, highlighting a need for enhanced governance and transparency [31].

### Key findings

High levels of corruption may have repercussions for health outcomes and fair financing in CEE (middle- to high-income settings), along with factors like government accountability, rule of law, health expenditure, and economic prosperity. While corruption has a smaller effect on health system outcomes compared to other economic and political factors considered, the indirect and insidious effect of corruption on population health and the health system remains problematic.

Lower rates of perceived corruption are associated with lower avoidable mortality, as are stronger government accountability and more robust application of the rule of law. Increasing GDP per capita has the most substantial effect on reducing avoidable mortality whereas, counterintuitively, a larger share of the government budget directed to the health system appears to increase avoidable mortality. Importantly, a higher proportion of the total population living in urban areas increases mortality from conditions amenable to healthcare.

Surprisingly, some of these same political measures are associated with higher OOP payments, although as noted, the data do not distinguish formal from informal payments. Lower rates of perceived corruption are associated with higher OOP payments, though an increase in GDP per capita has a greater effect in raising OOP payments. As expected, an increase in GHE results in a decline in OOP payments over time.

### Interpretation

Although corruption harms health, warranting targeted interventions, these results underscore the critical role of the quality of governance in fostering health system outcomes [27]. One possible interpretation is that lower corruption and higher government accountability may instil trust in the health system, prompting individuals to seek medical care in a timely fashion to address preventable and treatable conditions. Stronger application of the rule of law may also impact health system outcomes indirectly by encouraging adherence to public health policies that directly promote better health [32]. These effects support the argument for integrating Anti-Corruption, Transparency, and Accountability (ACTA) principles into health system strengthening efforts as a way of improving population health [2]. Yet while the quality of governance (QoG) is significantly associated with lower avoidable mortality in CEE, the adverse association with the Liberal Democracy Index warrants further investigation.

The effect of public health spending increases avoidable mortality in our analysis. This contradicts other research finding that healthcare expenditure lowers it, with past healthcare spending having a delayed diminishing effect on current avoidable mortality [33]. Nevertheless, there is growing evidence suggesting QoG partially mediates the impact of GHE on health outcomes [9], and that the effect of GHE on avoidable mortality changes over time [33]. This suggests while GHE may promote better health outcomes for certain countries and time frames [34], there are many factors affecting avoidable mortality and changing QoG in CEE may have mediating effects on GHE and GDP over time. The indirect positive impact of GDP on health system outcomes may be explained by the fact that both public and private health spending are correlated with national income, and the effect of increasing national income manifests into lower mortality rates over time [35].

This analysis indicates increasing urbanization raises avoidable mortality, which is counterintuitive given the evidence that urban populations have better health outcomes due to access to better-resourced care [32]. A possible explanation may be that urbanization tends to exacerbate socioeconomic inequalities in health and can amplify the adverse effects of material deprivation on mortality [36].

As the perception of corruption improves, the increase in OOP payments may be explained by the fact that countries with better governance often have higher OOP payments due to the formalization of direct health payments [37]. In line with previous findings, an increase in GHE is associated with a decline in OOP payments but as countries prosper, the use of formal private health services paid for by direct payments seems to grow [38]. The results corroborate existing evidence on the significant negative relationship between public health expenditure and OOP payments [39]. Increased GHE may reduce financial pressure by way of lower OOP payments, as expected, though if it is to reap the full potential of any additional public health investment, it must be coupled with governance measures enabling accountability and improving transparency [12].

The reported gaps in coverage in many CEE countries and variable quality of care may partly explain why citizens with more disposable income may opt for private health services via direct payments [38]. Thus, the measure we were able to use is not an ideal one and catastrophic spending would be better if it was available.

### Limitations

When interpreting the results, it is essential to acknowledge certain methodological constraints. The CPI relies on expert perceptions of corruption, and it is unclear if this reflects population perceptions [40]. Thus, a country’s annual score fluctuations might stem from evolving perceptions or methodological or sampling changes. Nevertheless, healthcare corruption has been shown to correlate with broader public sector corruption perceptions [15], and the CPI is widely seen as the best measure available to investigate the effect of corruption across time and countries.

Any association between corruption and health system outcomes is likely to be complex and multifaceted, with potential biases arising from unobserved variables, such as cultural or religious composition, though many of these are addressed by the use of a fixed-effects model. Corruption might also be linked to health; those in poorer health could be more inclined to offer bribes for essential services.

### Future research directions

Future studies should delve deeper into the interplay of culture, societal norms, and determinants of health concerning corruption, and its impact on key health indicators [7].

### Policy recommendations

We propose three key strategies that may be considered when strengthening the health systems in CEE countries. First, corruption hampers efforts to achieve UHC, and given the persistence of corruption and OOP payments, which are interrelated in complex ways, integrating ACTA measures into health systems is imperative. A systems-thinking approach, which considers power dynamics is crucial for the success of ACTA strategies.

Maintaining sufficient public health expenditure remains key. Results show that increasing public health expenditure significantly reduces OOP payments, though this does not necessarily eliminate informal payments, which persist as a subset of OOP payments. With an ageing European Union population and ageing and emigrating CEE workforces, there is an amplified risk of financial strain in healthcare.

Objective measurements of health sector corruption over time are needed but this requires a recognition that it still exists. There is a pressing need for improved data on catastrophic health expenditure especially given persisting informal payments. Digitized and institutionalized reporting mechanisms can generate evidence, promote transparency, and pinpoint areas for improvement.

## Conclusion

This analysis emphasizes the critical need for mobilizing global efforts against health sector corruption, even in regions with formally universal coverage such as the CEE countries. Our findings support the hypothesis that corruption may negatively affect health system outcomes, and this may occur both directly through the health system and indirectly, through wider political and economic forces. As many of the CEE countries advance towards UHC, the nuances of their financing mechanisms demand scrutiny, particularly considering governance quality. Sustaining investment into health systems is insufficient; good governance is paramount. Our study corroborates the perils of depending on direct payments in health financing, which intensifies inequities. Augmenting the public health expenditure's share in total health expenditure is pivotal for reducing OOP payments in CEE, with QoG playing a key role in mediating this relationship.

## Supplementary Material

ckae218_Supplementary_Data

## Data Availability

The authors confirm that all data supporting the findings of this study are publicly available [14, 24, 26, 29, 30]. The dataset used and analysed for this study is available from the corresponding author upon reasonable request. Perceived corruption is associated with higher avoidable mortality and greater out-of-pocket payments in Central and Eastern European countries. Corruption harms health system outcomes and warrants its own targeted interventions, though the quality of governance and other economic and social factors outside the health system also need addressing in interconnected ways to improve health outcomes and system functioning. Prioritising government accountability and effectiveness, and integrating Anti-Corruption, Transparency, and Accountability (ACTA) principles is vital for improving population health and financial protection. Monitoring health system functioning and evaluating health outcomes in countries with near-universal health coverage (UHC) remains essential. Future efforts should prioritize data collection and monitoring of corruption across sectors, as well as institutionalizing ACTA strategies, empowering grassroots actors, and digitizing governance.
