# Supplementary material for: Investigating the relationship between corruption and health system outcomes in Central and Eastern Europe
Source: Eur J Public Health. 2025 Oct 22;35(6):1076–81. doi: 10.1093/eurpub/ckae218 (PMC12707485; doi:10.1093/eurpub/ckae218)
Supplement: ckae218_Supplementary_Data [file ckae218_supplementary_data.docx]

# Supplementary Appendices: Investigating the relationship between corruption and health system outcomes in Central and Eastern Europe

Authors: Dina Balabanova ^1, 2,^ Petra Varkonyi ^1^, Martin McKee ^1, 3^, Darius Erlangga ^1^

1 London School of Hygiene & Tropical Medicine, London, UK

2 Department of Global Health and Development

3 Department of Health Services Research and Policy

Correspondence: Dina Balabanova, London School of Hygiene & Tropical Medicine, 15-17 Tavistock Place, London WC1H 9SH, United Kingdom, email: [dina.balabanova@lshtm.ac.uk](mailto:dina.balabanova@lshtm.ac.uk)

*Table S1: Outcome variables considered for the analysis and the rationale for selection.*

| ***Avoidable Mortality*** | |
| --- | --- |
| *Rationale for selection* | Central indicator of healthcare effectiveness. Captures deaths that should not occur in the presence of timely and quality care, as high levels of avoidable mortality and “slow or no improvement point to barriers in access or weaknesses in quality of care” [1]. The measure can help to illuminate inequalities in access to care within populations [2]. Among EU countries, avoidable mortality was highest in CEE countries at the turn of the century [3]. |
| *Indicator specifics* | Includes preventable and treatable mortality. Data are age-standardized and represent the mortality rate per 100,000 persons aged less than 75 years [4]. |
| *Limitations* | Issues with comparability of data, attribution of causes, and coverage of the range of health outcomes [2]. Country differences in diagnostic patterns or coding of cause of death may affect the level of recorded avoidable mortality rates across the region. |
| ***Out-of-pocket (OOP) Payments*** | |
| *Rationale for selection* | Strengthening health financing is one of the core objectives of SDG 3, recognizing that countries which rely heavily on OOP payments, including informal payments, to finance total health expenditures are unlikely to achieve UHC [5]. |
| *Indicator specifics* | Represents the share of OOP payments of total current health expenditures; OOP payments are spending on health directly out-of-pocket by households [6]. |
| *Limitations* | Indicator does not distinguish between the different types of direct payments. |

*Table S2: Potential confounding variables of corruption and health.*

| ***Variable Name*** | ***Description*** | ***Source*** |
| --- | --- | --- |
| ***Political Factors*** |  |  |
| Liberal Democracy Index | The liberal principle of democracy emphasizes the importance of protecting individual and minority rights against the tyranny of the state and the tyranny of the majority. | V-DEM Dataset |
| Rule of law | The extent to which laws are transparently, independently, predictably, impartially, and equally enforced, and the extent to which the actions of government officials comply with the law. | V-DEM Dataset |
| Accountability Index | Government accountability is understood as constraints on the government’s use of political power through requirements for justification for its actions and potential sanctions. | V-DEM Dataset |
| ***Economic Factors*** |  |  |
| Domestic general government health expenditure (GHE) | Public expenditure on health from domestic sources per capita expressed in international dollars at purchasing power parity. | WHO Global Health Expenditure database |
| GDP per capita, PPP (current international $) | Sum of gross value added by all resident producers in the country plus any product taxes and minus any subsidies not included in the value of the products. | World Development Indicators |
| Unemployment (% of total labour force) | Unemployment refers to the share of the labour force that is without work but available for and seeking employment. | World Development Indicators |
| ***Social Factors*** |  |  |
| Gini Index | Gini index of 0 represents perfect equality, while an index of 100 implies perfect inequality. | World Development Indicators |
| Urban population | % of total population living in urban area. | World Development Indicators |

*Table S3: Summary statistics of variables used (start and end time point values).*

| **Variable** |  | **N** | **Mean** | **Std. dev.** | | **Min** | **Max** |
| --- | --- | --- | --- | --- | --- | --- | --- |
| ***Outcomes*** |  |  |  |  |  | |  |
| Avoidable Mortality | 2012 | 12 | 460.61 | 92.87 | 297.41 | | 591.80 |
|  | *2020* | *12* | *451.10* | *95.60* | *268.30* | | *593.20* |
| Out-of-pocket payments | 2012 | 12 | 26.07 | 10.87 | 11.47 | | 47.75 |
|  | *2020* | *12* | *22.72* | *9.13* | *10.45* | | *36.58* |
| ***Political Factors*** |  |  |  |  |  | |  |
| Corruption Perception Index | 2012 | 12 | 50.50 | 7.93 | 39.00 | | 64.00 |
|  | *2021* | *12* | *52.33* | *10.10* | *38.00* | | *75.00* |
| V-Dem Liberal Democracy Index | 2012 | 12 | 0.71 | 0.12 | 0.49 | | 0.83 |
|  | *2020* | *12* | *0.62* | *0.18* | *0.25* | | *0.84* |
| V-Dem Accountability | 2012 | 12 | 1.46 | 0.27 | 1.01 | | 1.83 |
|  | *2020* | *12* | *1.20* | *0.47* | *0.25* | | *1.83* |
| V-Dem Rule of Law | 2012 | 12 | 0.83 | 0.13 | 0.57 | | 0.98 |
|  | *2020* | *12* | *0.80* | *0.15* | *0.49* | | *0.98* |
| ***Economic Factors*** |  |  |  |  |  | |  |
| GHE per capita, PPP (current intl $) | 2012 | 12 | 1120.20 | 393.04 | 623.50 | | 1794.78 |
|  | *2020* | *12* | *1889.34* | *645.17* | *1008.81* | | *3362.06* |
| GDP per capita (current intl $) | 2012 | 12 | 22972.75 | 4729.55 | 13933.83 | | 29254.73 |
|  | *2020* | *12* | *33885.29* | *6741.90* | *19557.58* | | *42827.06* |
| Unemployment | 2012 | 12 | 12.36 | 4.71 | 6.79 | | 24.00 |
|  | *2020* | *12* | *5.99* | *2.11* | *2.55* | | *9.01* |
| ***Social Factors*** |  |  |  |  |  | |  |
| Gini Index | 2012 | 12 | 32.48 | 4.57 | 25.60 | | 39.90 |
|  | *2019** | *12* | *30.9* | *5.14* | *23.2* | | *40.3* |
| % Urban population | 2012 | 12 | 62.62 | 7.89 | 53.11 | | 73.20 |
|  | *2020* | *12* | *63.70* | *8.29* | *53.76* | | *75.69* |

**2019 data presented (2020 data missing for Poland and Slovakia).*

*Table S4: Bivariate regressions for the outcome variables and each covariate.*

For avoidable mortality (column I), all predictors showed a significant correlation except for the rule of law and unemployment rate indicators. For OOP payments (column II), every predictor showed a significant correlation.

| **Predictors** | **N** | **Avoidable Mortality (I)** | | **Out-of-pocket Payments (II)** | |
| --- | --- | --- | --- | --- | --- |
|  |  | **Effect Size** | **P-value** | **Effect Size** | **P-value** |
| Corruption Perception Index (CPI) | 108 | -2.57 | 0.007 | -0.344 | 0.002 |
| Liberal Democracy Index | 108 | -111.21 | 0.039 | -26.708 | 0.001 |
| V-Dem Accountability | 108 | -47.76 | 0.03 | -9.547 | 0.001 |
| V-Dem Rule of Law | 108 | -82.47 | 0.165 | -21.989 | 0.002 |
| Log GHE per capita, PPP (current intl $) | 108 | -133.48 | 0.001 | -20.958 | 0.001 |
| Log GDP per capita (current intl $) | 108 | -92.51 | 0.003 | -21.856 | 0.001 |
| Unemployment | 108 | 2.27 | 0.252 | 0.660 | 0.005 |
| % Urban population | 108 | 2.42 | 0.023 | 0.536 | 0.001 |
| Gini Index | 106 | 10.64 | 0.001 | 1.625 | 0.001 |

*Table S5:* *Sensitivity Analysis - fixed effects panel data analysis of each health outcome (excluding 2020).*

|  | **Avoidable Mortality** | | | **Out-of-pocket Payments** | | |
| --- | --- | --- | --- | --- | --- | --- |
|  | Effect Size | 95% Confidence Interval | P-value | Effect Size | 95% Confidence Interval | P-value |
| **Corruption Perception Index** | -1.72 | [-3.05, -0.40] | 0.012 | 0.18 | [0.02, 0.34] | 0.027 |
| **Accountability Index** | 13.36 | [-77.97, 104.70] | 0.772 | 2.47 | [-8.27, 13.22] | 0.648 |
| **Rule of Law** | -158.14 | [-253.19, -63.08] | 0.001 | -3.27 | [-14.57, 8.03] | 0.566 |
| **Liberal Democracy Index** | -20.92 | [-249.83, 207.99] | 0.856 | -2.29 | [-29.35, 24.77] | 0.867 |
| **GHE per capita (current intl $)** | -18.80 | [-92.06, 54.47] | 0.611 | -10.43 | [-19.33, -1.54] | 0.022 |
| **GDP per capita (current intl $)** | -132.32 | [-241.19, -23.44] | 0.018 | 6.62 | [-7.34, 20.58] | 0.348 |
| **Gini Index** | 0.02 | [-2.44, 2.48] | 0.987 | -0.10 | [-0.39, 0.20] | 0.513 |
| **Unemployment** |  |  |  | 0.02 | [-0.23, 0.27] | 0.868 |
| **% Urban population** | 3.58 | [-7.65, 14.82] | 0.527 | 0.58 | [-0.75, 1.92] | 0.387 |
| **Constant** | 1900.32 | [1429.04, 2371.61] | 0.000 | -9.89 | [-88.0, 68.21] | 0.801 |
| **Number of observations** | 96 | | | 96  Within = 0.2204  Between = 0.2823  Overall = 0.2811 | | |
| **R-squared** | Within = 0.7593  Between = 0.1200  Overall = 0.1490 | | |  |  |  |
|  |  |  |  |  |  |  |
|  |  |  |  |  |  |  |

# References

1. Papanicolas I, et al., *Health system performance assessment: a framework for policy analysis*. Health Policy Series 57. European Observatory on Health Systems and Policies, 2022

2. Nolte E and McKee M, *Does Health Care Save Lives? Avoidable Mortality Revisited*. 2004

3. Newey C, et al., *Avoidable Mortality in the Enlarged European Union*. 2004: Technical Report. Institut des Sciences de la Sante, Paris.

4. Eurostat. *Standardised preventable and treatable mortality*. 2023; [https://ec.europa.eu/eurostat/databrowser/view/sdg_03_42/default/table:[Available](https://ec.europa.eu/eurostat/databrowser/view/sdg_03_42/default/table:%5bAvailable) from: <https://ec.europa.eu/eurostat/databrowser/view/sdg_03_42/default/table>.

5. Vian T, et al. Barriers to universal health coverage in Republic of Moldova: a policy analysis of formal and informal out-of-pocket payments*.* *BMC Health Serv Res* 2015; **15**: 319.

6. *World Development Indicators*. 2023, The World Bank: Washington D.C. <https://databank.worldbank.org/source/world-development-indicators>.
